# Observations on Baron Larrey's Paper Concerning the Reproduction of the Vitreous Humour*

**Published:** 1817-06

**Authors:** G. Beauchamp Knowles

**Affiliations:** Member of the Royal College of Surgeons in London.


### For the London Medical and Physical Journal.

*Observations on Baron Larrey's Paper* concerning the Reproduction of the Vitreous Humour;* by G. BEAUCHAMP KNOWLES, Member of the Royal College of Surgeons in London.

HAVING noticed, in your Journal for March last, a case of wounded cornea followed by the loss of a portion of the vitreous humour, I beg leave to offer a few observations upon it, which I am induced to do chiefly because Baron Larrey not only seems to consider it an extraordinary instance of recovery, but also draws conclusions from it, which even its fortunate termination does not seem to warrant. I allude to the case of M. Dreux, whose wound, according to his own account, was instantly followed by the evacuation of a thick limpid liquor, and the flattening of the eye. He abandoned all idea of ever recovering the sight of his eye; but, to my great surprise, (says Baron Larrey,) the globe gradually resumed its primitive form and natural size;" and he then adds, "so that it cannot be doubted that the vitreous humour, of which a certain portion had really exuded, was regenerated; and this fact proves that Nature can regenerate it wholly, or, at least, in part."

I would here observe, that, though the eye is frequently reduced in size after such an accident, yet it is well known that such is not always the case; neither is it always followed by a deprivation of sight. In short, numerous well-authenticated cases prove the fact, that the sight may be preserved, notwithstanding a very large effusion of the vitreous humour; and of this I have seen frequent instances. Baron Wenzel, in his treatise on the Cataract, relates many cases where a portion of the vitreous humour escaped during the operation, without being followed by any ill consequences. In one case he says, "the loss of this humour, as nearly as I could judge, was equal to three-fourths of its whole quantity; and, though I had often seen considerable portions of it discharged without destroying the sight, yet, in this case, the quantity that escaped was so considerable, that I could not refrain from giving up the eye as entirely lost." At the end of three days, however, she distinguished every object she looked at. He admits that the eye was much reduced in size, and the pupil greatly dilated; but he concludes his account of the case by stating, that she after-

* See the case of M. Dreux, in our present vol. p. 215.

wards wards enjoyed as good a sight as is ever experienced after the most successful operation.

With respect to the re-production of the vitreous humour in M. Dreux's case, and which Baron Larrey thinks "cannot be doubted," I must confess I entertain very strong doubts upon the subject. Let us for a moment reflect upon the structure of the vitreous humour, which I consider (though contrary to the opinion of some anatomists) to be contained in two transparent membranes or tunics, denominated Tunica hyaloidea externa et interna. The former envelops the whole of the vitreous humour, so called; the latter forms numerous delicate cellulæ, which communicate with each other, and are filled with a liquor not essentially different from the aqueous humour. Now, when a portion of such cellulæ is separated and expelled from the eye, is it reasonable, I would ask, to suppose that they are ever re-produced? Do we ever see parts re-produced in their original state of perfection? I believe, never; for, though it has been ascertained by experiment, that nature is able to regenerate the eye of a newt, or the claw of a lobster, yet I think I may venture to say, that we have no instance of the perfect re-production of a destroyed part in the human body. If a muscle be divided, it is united by a tendinous, and not by a muscular, substance. If a portion of cornea slough, nature may repair the breach, but the newly-formed part is never transparent. I am inclined to think, therefore, that the vitreous humour is never perfectly regenerated, but that its loss is supplied by the aqueous humour; and that the chance of the globe remaining diminished in bulk, or resuming its primitive size, will depend, partly upon the degree of consequent inflammation, and partly upon the rapidity with which the aqueous humour is secreted.

*Birmingham; April* 11, 1817.

